# ChatGPT in Pediatric Dental Trauma: A Cross-Sectional Assessment of Response Reliability to Parental Queries

**DOI:** 10.7759/cureus.111074

**Published:** 2026-06-18

**Authors:** Ruhi Sanvordekar, Elaine S Barretto, Dinesh F Swamy, Aswathy Sudarsanan

**Affiliations:** 1 Department of Pediatric and Preventive Dentistry, Goa Dental College and Hospital, Bambolim, IND

**Keywords:** artificial intelligence (ai), chatgpt, clinical pediatric dentistry, pediatric dental trauma, traumatic dental injuries (tdis)

## Abstract

Aim and background

Parents often turn to the Internet for answers regarding their child’s health due to limited access to doctors on-demand and curiosity about medical conditions or treatments. With its human-like interaction and artificial intelligence (AI), ChatGPT (OpenAI, San Francisco, California) is a popular AI-supported tool that is expected to become commonly used. Traumatic dental injuries (TDIs) are common in children, yet parents are often unaware of appropriate aid. Since timely emergency care significantly affects treatment outcomes, many parents may seek advice from AI chatbots during such situations. This study aimed to assess the relevance, correctness, logic, clarity, and completeness of ChatGPT’s responses to frequently asked parental questions following dental trauma scenarios.

Methods

This study was conducted at Goa Dental College and Hospital, Bambolim. A set of 15 questions from a parent-perspective depicting scenarios were drafted and entered into ChatGPT version 4.0. A panel of 29 experts evaluated the responses using a pre-validated questionnaire, rating each answer on a five-point scale across five parameters, namely, relevance, correctness, logic, clarity, and completeness, along with an open-ended question to determine what the experts would have added to the ChatGPT response.

Results

The mean score across all responses was 4.35, indicating that most ChatGPT answers were of good quality with minimum inaccuracies. Completeness had the lowest score among all parameters, with only 82.76% of respondents rating it ≥ 4 (score 4 = adequate), indicating that there were some missing components in the answers.

Conclusion

Answers were mostly found to be relevant, logical, correct, complete, and in clear language.

Clinical significance

ChatGPT can serve as a helpful tool for parents seeking timely, evidence-based information about dental trauma in children. This research is a foundational step for early evidence on the reliability of ChatGPT in the dental domain.

## Introduction

Parents frequently access different Internet sources and social media platforms for answers to their medical queries. Due to the inability to talk to doctors whenever they want, inability to find answers in an easy manner, and curiosity regarding experiences of individuals with similar medical histories, in earlier days, people used to try to find answers from their peers and elders and through other resources, like printed and electronic media, books, magazines, and interviews with doctors. In the last decade, there has been a steep increase in the possession and use of smartphones along with easy access to the Internet. However, to find answers to their medical queries, parents have to scroll through many search results and websites and may get attracted to “fancy” websites or those which provide biased and sponsored content [1]. Despite availability and easy access to information, the quality of retrieved information has been critiqued in various studies by Aksoy et al. [2] and Varghese et al. [3] to be of poor to medium quality, having low reliability, incomplete, and sometimes even wrong. There are no in‑built checks to filter out the correct and evidence‑based information on these search engines that patients are likely to visit [1].

In recent years, there has been a surge in the popularity and availability of artificial intelligence (AI) tools that can learn, adapt, gain experience, perform human-like interactions, and mimic human intelligence to perform tasks. Unlike search engines that retrieve and provide links to information on a given topic, AI-based chatbots engage in conversational interactions and provide authoritative-sounding responses to medical queries, making it easier and more convenient for people to understand complex topics [4,5].

One such AI tool that has gained widespread popularity recently is Chat Generative PreTrained Transformer (ChatGPT), a large language model (LLM) released on November 30, 2022, by OpenAI (San Francisco, California). Despite its potential, caution has been warranted when using it in medical practice as the reliability and accuracy of this platform have not been assessed, particularly with open-ended medical queries that patients are likely to ask. The literature on the adoption of ChatGPT and similar natural language processing (NLP) AI tools suggests that the use of these would soon become a common place and is expected to be frequently used by patients and parents for answers to their queries, as if conversing with a doctor [1].

Pediatric dental experts routinely address parental concerns and are therefore well positioned to judge whether information aligns with parental expectations in terms of clarity, relevance, and practical usefulness. Their evaluations act as a surrogate measure of parental perspectives, whether the information provided meets the concerns and decision-making requirements of parents following dental trauma to their child. Thus, expert judgement offers an indirect yet clinically meaningful measure of how well AI-generated responses align with parental expectations. 

Dental traumatology is a specialized field of dentistry that deals with the study and management of traumatic injuries affecting the teeth, jaws, and adjacent oral structures, encompassing aspects, such as their causes, distribution, prevention, evaluation, diagnosis, and treatment [5]. The treatment options in dental trauma are diverse and the timing of treatment is as crucial as the treatment itself, as most of the negative outcomes are due to inadequate or inappropriate first-aid interventions [5]. Hence, immediate access to accurate and comprehensible information is essential, as delays or inappropriate first-aid measures may adversely affect prognosis [6]. Recent studies have explored the role of LLMs in dental traumatology and have demonstrated promising performance in providing guideline-based information related to traumatic dental injuries (TDIs). However, concerns regarding variability in accuracy, completeness, clarity, and readability of AI-generated responses continue to exist, highlighting the need for further validation in clinically relevant scenarios [7,8,9]. Hence, the primary objective of this study was to evaluate the expert-perceived quality of ChatGPT-generated responses to frequently asked questions from a parent's perspective following pediatric dental trauma. Specifically, the study assessed the relevance, correctness, logic, clarity, and completeness of ChatGPT responses. A secondary objective was to identify inaccuracies and missing information in ChatGPT responses that experts considered important for parental guidance.

## Materials and methods

This study was conducted at Goa Dental College and Hospital, Bambolim, India. Ethical approval for the study was obtained from the Institutional Review Board (GDCH/IRB/VIII-2024/RPN50-48). The sample size was calculated based on data procured from a former study by Gugnani et al. [1]. For an α error value equal to 0.05 and a significance level of 1.96, the sample size was calculated to be 29. Participants included experts in the field of Dental Traumatology selected on an arbitrary basis. The experts chosen had varied experience in clinics, academics, and research in Pediatric Dentistry and Endodontics.

This study was a descriptive, cross-sectional, vignette‑based survey carried out between August 2024 and September 2024. A set of 15 questions was drafted by the authors, depicting scenarios as if being asked by a parent following dental trauma to their child (Table 1).

**Table 1:** List of scenario-based questions presented to ChatGPT

Q. No.	Question presented to ChatGPT
1	My child had a fall and his front milk tooth has been knocked out. What should I do?
2	My child had a fall and his front permanent tooth has been knocked out. What should I do?
3	How should I carry a knocked out tooth or broken tooth piece to the dentist?
4	My child had a knocked out tooth in school but has come home with the tooth now. Can I put my child's knocked-out tooth back in place?
5	My child had a fall and now one of his front tooth is loose and shaking. What should I do?
6	My child had multiple loose teeth after a fall which were fixed by a dentist. What precautions should I take at home after this procedure?
7	My child hit his teeth while playing and one of his permanent tooth has partially gone inside the gums. The dentist did not do any treatment and told me tooth will come in place on its own. Is it correct or do I have to be concerned?
8	My child hit his teeth while playing and one of his milk tooth has partially gone inside the gums. What will the dentist do?
9	After a fall, my childs front tooth has partially come out below the level of surrounding teeth. What is the treatment for this?
10	Can a broken tooth piece be glued back by a dentist?
11	My child had hit his mouth while playing 20 days back. Now I am noticing his front tooth becoming darker in colour. What is the treatment for this?
12	My child has fallen down and is bleeding from her cut lower lip. The bleeding has not stopped since the last 15 minutes. What should I apply to stop the bleeding?
13	My child fell down and has a damaged milk tooth. Is it okay if I don’t get it treated? Or will it have any effect on his permanent tooth?
14	What should I keep in my first aid kit at home in case my child falls down and damages his teeth?
15	Is there anything that can be done to prevent injuries to teeth during sports?

**Figure 1:**
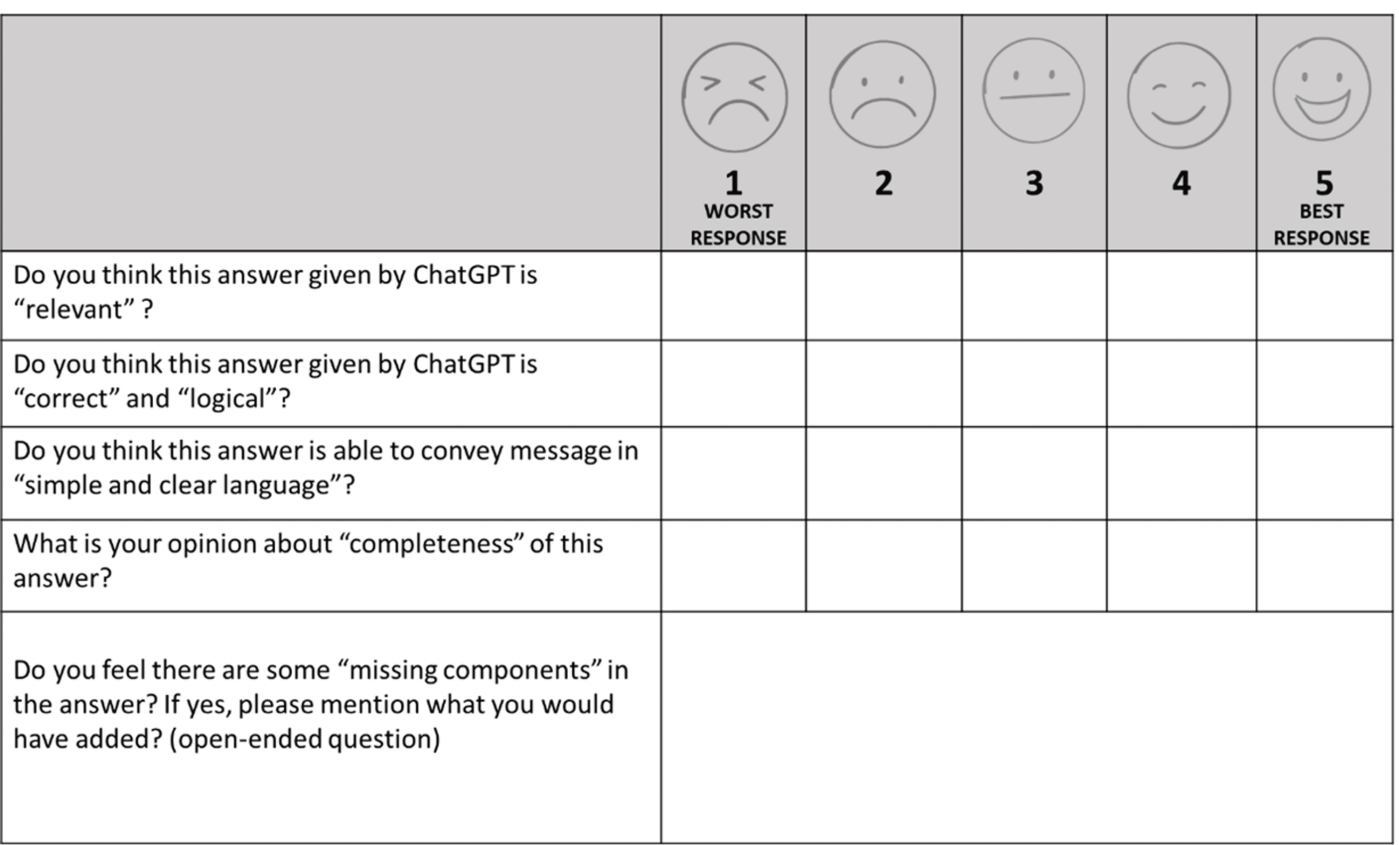
Questionnaire to assess ChatGPT answers

To ensure the quality and appropriateness of these questions, face validity was assessed by a panel of three pediatric dental specialists, who evaluated each question for clarity, realism, and relevance to everyday clinical situations. Based on their feedback, minor modifications were made to improve phrasing and ensure alignment with typical parental perspectives. Each question was entered into ChatGPT version 4.0 in a single session. The corresponding response by ChatGPT was exported into Microsoft Word (Microsoft Corp., WA, USA) along with a pre-validated questionnaire [1]. This questionnaire included questions about the relevance, logic, correctness, clarity, and completeness of the ChatGPT‑generated answers (Figure 1).

These questions were based on the Likert scale, in which respondents had to answer on a scale of one to five, with one being the “worst response” and five being the “best response." In addition, there was an open‑ended question aiming to determine what participants would have added to each response as an expert in the field. The questionnaire was sent to all the experts, and they were provided one week to respond (see Appendix 1).

**Figure 2:**
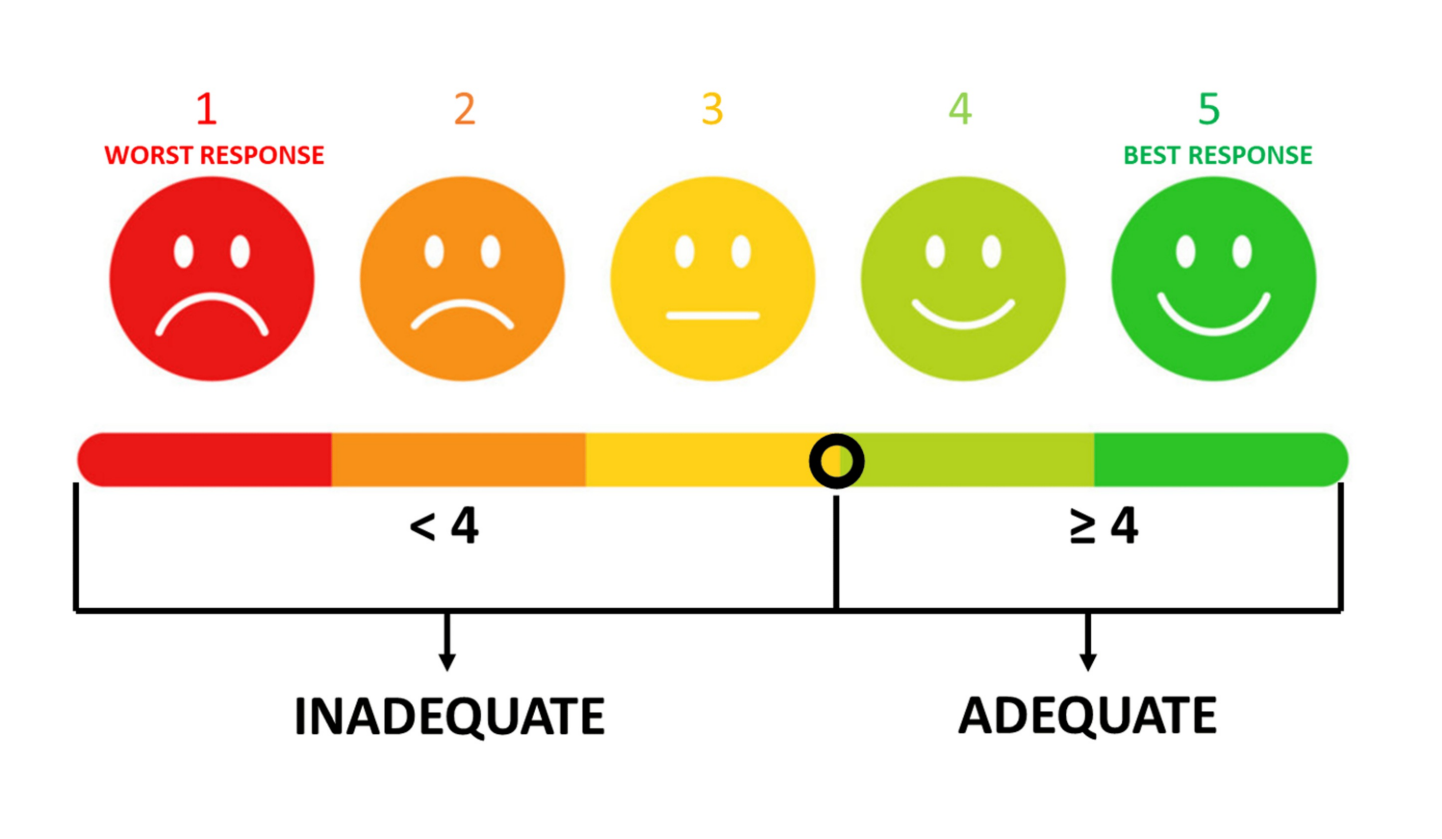
Interpretation of the scoring data

The scoring data from participants’ responses as per Likert’s scale was condensed as score of ≥4 = adequate and acceptable and score of <3 = inadequate (Figure 2).

Data were summarised using descriptive statistics on IBM SPSS Statistics for Windows, version 21.0 (released 2012, IBM Corp., Armonk, NY).

## Results

In accordance with the study objective of evaluating the quality of ChatGPT responses, the overall mean score across all assessed parameters was 4.35, indicating generally favourable expert evaluations. The number and percentage of respondents who scored more than or equal to 4 on the Likert scale were determined across all questions and parameters (Table 2).

**Table 2:** Number and percentage of respondents who marked equal to or more than 4

Q no	Relevance	%	Logic	%	Clarity	%	Completeness	%
1	27	93.1	23	79.31	25	86.21	23	79.31
2	28	96.55	26	89.66	29	100	22	75.86
3	26	89.66	26	89.66	29	100	26	89.66
4	20	68.97	18	62.07	22	75.86	20	68.97
5	26	89.66	28	96.55	28	96.55	26	89.66
6	29	100	29	100	29	100	28	96.55
7	25	86.21	24	82.76	28	96.55	23	79.31
8	24	82.76	26	89.66	26	89.66	26	89.66
9	29	100	29	100	28	96.55	22	75.86
10	28	96.55	29	100	28	96.55	26	89.66
11	28	96.55	24	82.76	27	93.1	23	79.31
12	27	93.1	28	96.55	29	100	24	82.76
13	25	86.21	24	82.76	25	86.21	22	75.86
14	25	86.21	25	86.21	26	89.66	23	79.31
15	26	89.66	23	79.31	26	89.66	26	89.66

The mean percentage of respondents who marked more than or equal to 4 on relevance was found to be 90.34%, for logic was 87.8%, for clarity was 93.1%, and for completeness was 82.76%.

**Figure 3:**
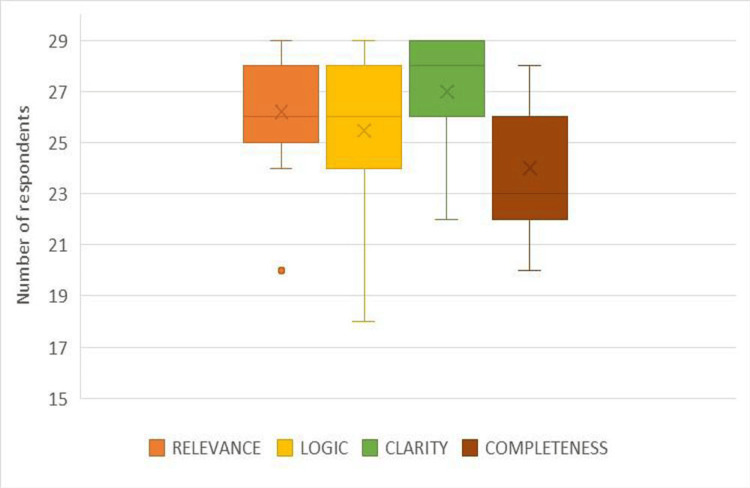
Box plot showing the number of respondents rating ChatGPT responses across four quality parameters

A box plot was plotted depicting the number of dentists who responded ≥4 for different questions (Figure 3).

The number of dentists who marked ≥4 on relevance on all 15 questions was concentrated around 25-28 dentists, with a median of 26. The number of dentists who marked ≥4 on logic on all 15 questions was concentrated around 24-28 dentists, with a median of 25.5. The number of dentists who marked ≥4 on clarity on all 15 questions was concentrated around 26-29 dentists, with a median of 27. The number of dentists who marked ≥4 on completeness on all 15 questions was concentrated around 22-26 dentists, with a median of 24.

The responses for the open‑ended question on missing components that respondents would have added if a patient had approached them with the query were summarized after de‑duplication, and similar points by different respondents were merged (Table 3).

**Table 3:** Summary of expert remarks on missing components or inaccuracies in ChatGPT responses

Q. No.	Missing components/remarks by respondents
1	• Ensure whether milk tooth or permanent tooth
	• Emphasis required on do not attempt to reinsert the milk tooth by yourself
	• Possibility of the dentist reimplanting the milk tooth if the factors are favorable
	• Pain management could have been mentioned.
2	• Do not rinse the tooth with "lukewarm" water.
	• While rinsing the tooth in the sink, make sure the valve of the sink is not open.
	• Attempt to reinsert the tooth not mentioned
	• Instructions regarding the tetanus vaccine not mentioned
	• The ideal temperature for the storage of milk should have been mentioned – cold or room temperature, not hot or boiling.
	• Root canal treatment will be done or revascularisation treatment based on the age of the child and root development – what they can expect from the dentist.
3	• Do not rinse the tooth with lukewarm water; rinse with saline or milk.
	• Although water is a poor medium, it is better than leaving the tooth to air-dry.
	• More storage media – HBSS could have been mentioned.
	• The term "jostling" could have been mentioned with a simpler term like "shaking."
4	• Attempt reinsertion only if very sure it is a permanent tooth; milk teeth not to be reinserted.
	• Timing between trauma and reinsertion
	• Do not force/push the tooth in the socket while trying to reinsert if there is resistance.
	• If the patient is unconscious, do not attempt to reimplant the tooth.
	• After reinserting, ask the patient to bite on a gauge/handkerchief stabilisation of the tooth till the patient reaches the dentist – not mentioned.
	• Although water is a poor medium, it is better than leaving the tooth to air-dry.
	• Pain or discomfort while reinserting
5	• "Splinting" could have been explained in simple terms, like repair or bonding with a wire.
	• Time duration for splinting – a few to several weeks.
6	• Don’t try to wiggle/shake the tooth.
	• If front tooth, don’t bite/pull with front teeth.
	• Frequent concern of parents – to send child to school/not till tooth gets stabilised – not addressed
	• Emphasis on the importance of follow-ups
7	• Reassurance is missing – many teeth that are pushed inwards have a good chance of erupting back to their original position without the need for any treatment.
	• Soft diet for how long
	• Rinsing with antibacterial mouthwash
	• Time period after which the parent needs to revisit the dentist in the case of non-eruption of the tooth back to its original position
8	-----
9	• Differences in treatment for the extrusion of primary and permanent tooth – not mentioned
	• The possibility of the tooth becoming nonvital and requiring root canal treatment could have been mentioned.
	• Could give an average range of splinting time duration in weeks
10	------
11	• "Internal bleeding" – inappropriate term
	• Could have mentioned that the dentist will do a vitality test of the tooth to check if the nerves and blood vessels inside the tooth are destroyed
	• Dark colour does not stabilise/revert.
	• Methods to improve appearance: bleaching, veneers, crowns – not mentioned
12	• If the child has any known bleeding or clotting disorder, seek medical help immediately.
13	• Permanent tooth might get hypoplastic (white/brown spots) or other anatomical defects – negative effects/sequelae of not getting the milk tooth treated – not mentioned
14	• Not advisable to reattach a broken tooth piece with temporary cement or dental adhesive at home – risk of aspiration
	• Fresh milk is to be used for the storage of the tooth. A container for milk can be kept instead.
	• A list of mild antiseptic agents could have been given along with the concentration instead of saying avoid strong antiseptics.
15	• Certain individuals with forwardly placed teeth have an increased chance of injuries and can consider undergoing treatment to correct their teeth and profile.

## Discussion

In this study, we evaluated the ability of ChatGPT to answer some common parental queries following dental trauma to their child. Overall, we found that most responses were of good quality across parameters of relevance, logic, and clarity, with minimal inaccuracies and some amount of missing information. These findings are consistent with the growing body of literature assessing LLMs as adjunctive tools in dental information delivery.

Gugnani et al. [1] found that ChatGPT provided largely logical, clear, and comprehensive answers to maternal queries related to child oral health, with minimal misinformation. Jacobs et al. [4] reported that ChatGPT’s responses to patient questions on third molar extractions were largely accurate and closely aligned with established clinical guidelines. Similar findings were reported by Sağlam et al., who demonstrated that ChatGPT-4o showed significantly higher guideline-based knowledge and response accuracy regarding traumatic dental injury management compared to dentists and other AI models, further supporting the potential role of AI-assisted platforms in dental trauma education and emergency guidance [7]. Keleş et al reported that AI chatbots demonstrated high diagnostic and management accuracy in simulated dental trauma cases based on International Association of Dental Traumatology (IADT) guidelines, supporting the growing potential of AI-assisted systems as adjunctive tools in dental trauma decision-making and patient guidance [10]. Our findings corroborate these observations, particularly with respect to clarity and relevance, which received the highest expert ratings. 

However, variability in accuracy and completeness has been consistently highlighted in dental trauma-specific literature. Ozden et al. [5] compared ChatGPT and Google Bard (Gemini) for dental trauma-related questions and reported limitations in accuracy and consistency, particularly when responses required nuanced clinical judgment. While their study employed dichotomous questions, our vignette-based, open-ended approach better reflects real-world parental queries. Despite encouraging findings, previous investigations have emphasized that AI-generated responses may still contain inaccuracies, inconsistencies, or incomplete recommendations [9].

Kuru et al. [11] evaluated multiple AI platforms for dental trauma management and found significant inter-model variability, with no platform demonstrating consistently superior performance across all injury types. Çege et al. [12] assessed AI chatbots using clinical images and ToothSOS recommendations, reporting reduced accuracy for complex injuries. Their findings align with the inaccuracies and missing components identified by experts in our study.

The importance of guideline-driven and domain-specific AI tools has been emphasized by Luiz et al. [13], who demonstrated improved accuracy and completeness using a dental trauma chatbot explicitly programmed according to the IADT guidelines [14]. This suggests that unrestricted generative models, such as ChatGPT, may benefit from integration with validated clinical frameworks. Furthermore, Haupt et al. [15] highlighted that ChatGPT’s educational utility is highly dependent on prompt structure, a factor particularly relevant when parental queries are vague, emotionally driven, and time-sensitive.

From a caregiver-centered perspective, Gökcek and Nale [16] evaluated AI chatbot responses using patient-information quality metrics and concluded that while AI tools can support parental understanding, they should not replace professional advice. Our results support this conclusion, as expert evaluators identified missing information related to critical first-aid instructions, contraindications, and follow-up care that are essential for safe parental decision-making.

In our study, we found a few minor inaccuracies in the ChatGPT answers. In questions regarding the management of avulsion, ChatGPT's answer mentioned that an avulsed tooth should be washed with lukewarm water. However, "lukewarm" is a very subjective term and may be misinterpreted as rinsing with hot water, which may cause further damage to the periodontal ligament cells. Regarding storage media for an avulsed tooth, ChatGPT's answer mentioned that it should never be stored in water. However, according to the IADT guidelines [14], while water is not an ideal storage medium, it is preferable to allow the tooth to air-dry. In a question about loose teeth, ChatGPT used terms like "splinting," which could have been explained in simpler terms, like repair or bonding with a wire. A major inaccuracy was noted in a question inquiring about what a parent should keep in their first aid kit for dental trauma. ChatGPT's answer mentioned that a broken tooth piece can be reattached at home with temporary cement or dental adhesives, but this is not advisable and may prove to be hazardous due to the risk of aspiration. Another inaccuracy was noted that ChatGPT mentioned a container with milk can be kept in the kit for storage of tooth in case of avulsion injury, but instead of this, a container for milk can be kept, and fresh milk is to be used for storage. ChatGPT mentioned in an answer that a permanent tooth, if knocked out, should be attempted to be reinserted; however, it missed out on the fact that reinsertion of an avulsed tooth should never be carried out if the patient is unconscious. Moreover, ChatGPT's answer did not include biting on a gauge or handkerchief for stabilisation of the tooth till the patient reaches the dentist, as recommended by the IADT [14].

The percentage of respondents who marked completeness of ChatGPT answers as ≥4 was the lowest, i.e., 82.76%, among all the parameters, indicating that there were missing components in the answers. We compiled the responses by the panel of experts to the open-ended question regarding missing components and inaccuracies in ChatGPT answers (Table 3). Some of the important points missed out were that milk tooth should never be attempted to reinsert in a socket by the patient themself, the ideal temperature of milk for storage of avulsed tooth, soft diet to be given for how long after splinting, and the requirement of tetanus vaccination post trauma.

There are various limitations to this study. We tested a limited number, i.e., 15 questions or scenarios. We also tested only one AI platform, i.e., ChatGPT. A five-point Likert scale was used to assess the answers, which do not provide much information. Further studies should evaluate more AI platforms and explore more scenarios of different topics in dentistry. Variability in AI responses across different AI models and repeated interactions with the same model has been documented in recent literature, which may influence the consistency of generated recommendations [8]. Hence, evaluation of answers should be carried out on multiple occasions and by different individuals. This was another limitation of the present study; hence, further research needs to evaluate the consistency of this platform in giving accurate answers every time. A potential source of bias includes expert selection bias, as participants were recruited on a convenience basis. To minimise this, a panel comprising experts with diverse academic, clinical, and research experience in pediatric dentistry and endodontics was included. Despite the limitations, this study expands on the previous literature on ChatGPT in dental traumatology.

## Conclusions

ChatGPT responses to common parental queries regarding pediatric dental trauma were generally rated as relevant, logical, clear, and largely accurate by expert evaluators. Although rated favourably, several inaccuracies and missing components were identified. Therefore, ChatGPT should be used as an adjunctive resource for supplementary information rather than a substitute for professional dental assessment and advice. This study is a foundational step toward establishing early evidence base toward the reliability of ChatGPT in dentistry.

This study highlights the clinical significance of ChatGPT as a supportive tool for parents seeking timely and evidence-based information regarding dental trauma in children. By evaluating the reliability of ChatGPT’s responses to common pediatric dental trauma queries, the findings offer valuable insights for pediatric dentists. This can assist clinicians in guiding families on the cautious and informed use of ChatGPT for health-related concerns. The research serves as an important early contribution to the growing body of evidence on the applicability and reliability of AI in the dental domain.

## Figures and Tables

**Figure 4:**
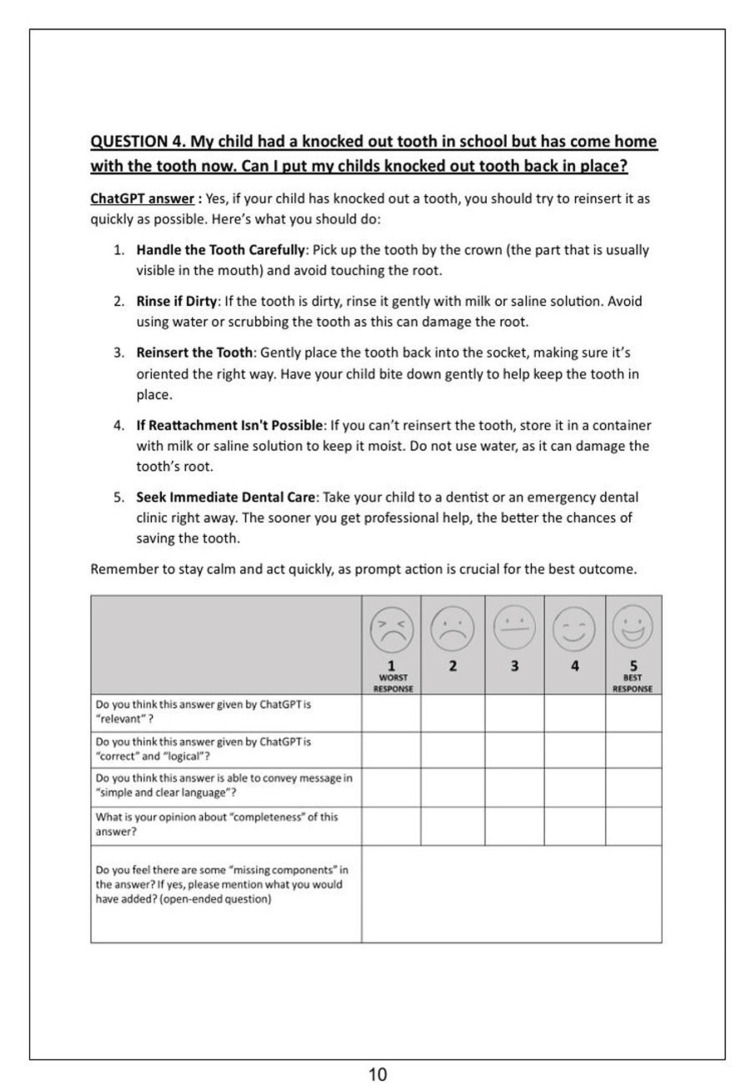
Sample sheet of the questionnaire provided to experts for evaluating ChatGPT-generated responses on dental trauma
